# Spontaneous Massive Intrathoracic Hematoma in a Patient with Marfan Syndrome

**DOI:** 10.3400/avd.cr.26-00097

**Published:** 2026-09-10

**Authors:** Yuma Yokoyama, Kanako Kobayashi, Takahito Itoh, Takashi Murakami

**Affiliations:** Department of Cardiovascular Surgery, Tokyo Saiseikai Central Hospital, Tokyo, Japan

**Keywords:** Marfan syndrome, intrathoracic hematoma

## Abstract

Although aortic complications are common in patients with Marfan syndrome (MFS), spontaneous peripheral arterial rupture is rare. Herein, we report a 40-year-old woman with MFS who presented with left-sided chest pain and hoarseness due to a massive intrathoracic hematoma. She had undergone multiple thoracic surgeries for aortic dissection. Rupture of fragile neovessels associated with surgery-related inflammation was suspected. Hemostasis was achieved by transcatheter embolization of the left lateral thoracic and supreme intercostal arteries. Intrathoracic bleeding should be considered in patients with MFS presenting with chest pain or hoarseness, particularly those receiving anticoagulation therapy or those who have undergone prior thoracic surgery.

## Abbreviations

MFS
: Marfan syndrome
PNALs
: primary non-aortic lesions
CT
: computed tomography

## Introduction

Marfan syndrome (MFS) is a connective tissue disorder that is typically caused by mutations in the *FBN1* gene.^1)^ This disorder is thought to involve multiple organ systems, including the cardiovascular, skeletal, and ocular systems, through microfibril dysfunction and dysregulated transforming growth factor-β signaling.^1)^

Cardiovascular complications, particularly aortic aneurysms and dissections, are major causes of morbidity and mortality in patients with MFS. Consequently, patients frequently require surgical intervention for aortic disease, making long-term imaging surveillance essential. Surveillance for non-aortic lesions, including aneurysms and ectasia, is also essential. Primary non-aortic lesions (PNALs) are peripheral arterial lesions that do not result from extension of aortic dissection. Sénémaud et al. reported that PNALs were identified in 20.3% of 138 patients with an *FBN1* pathogenic variant and that some aneurysmal lesions required surgical intervention.^2)^

Beyond these recognized vascular manifestations, a much rarer pattern of hemorrhage has been reported. In such cases, it has been suggested that fragile neovascularization arising from chest wall arteries forms abnormal collateral vessels extending into the lung, which subsequently rupture.^3,4)^ Here, we report a case of MFS with no clearly identifiable PNALs on computed tomography (CT) angiography in which a massive left intrathoracic hematoma subsequently developed.

## Case Report

A 40-year-old woman with MFS presented with left-sided chest pain and hoarseness. Eight months earlier, she had developed acute Stanford type A aortic dissection and severe aortic regurgitation and subsequently underwent multiple thoracic aortic surgeries. CT revealed a massive hematoma in the left thoracic cavity accompanied by a mild pneumothorax. No hemoptysis was observed.

Her surgical history included an emergent modified Bentall procedure, followed by total arch replacement and descending thoracic aortic replacement for a progressive dissecting aneurysm. She had marked scoliosis and was subsequently found to harbor an *FBN1* mutation, leading to the diagnosis of MFS. Postoperative CT showed no apparent abnormalities and no PNALs were identified.

**Fig. 1:**
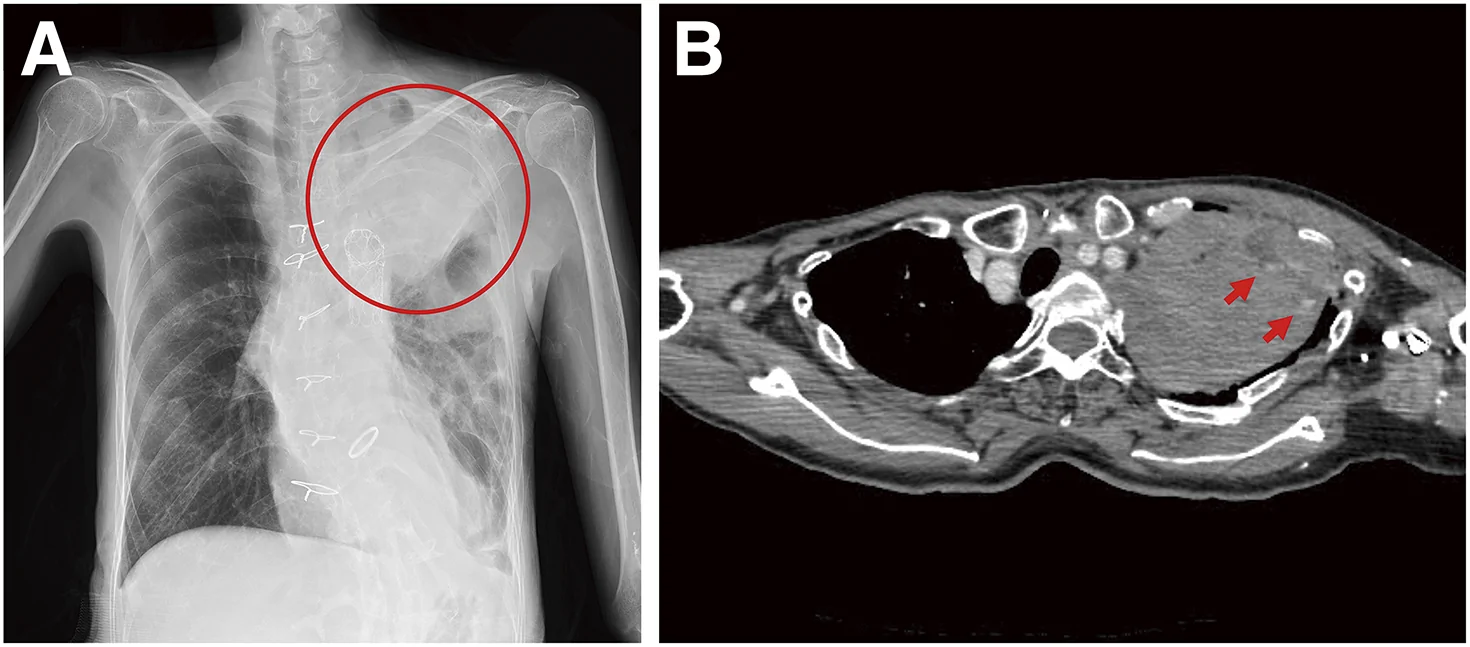
Imaging findings at admission. (**A**) Chest radiography showed a large opacity in the left hemithorax (circle). (**B**) Contrast-enhanced CT revealed a massive hematoma in the left thoracic cavity, with focal contrast enhancement (arrows) within the hematoma.

**Fig. 2:**
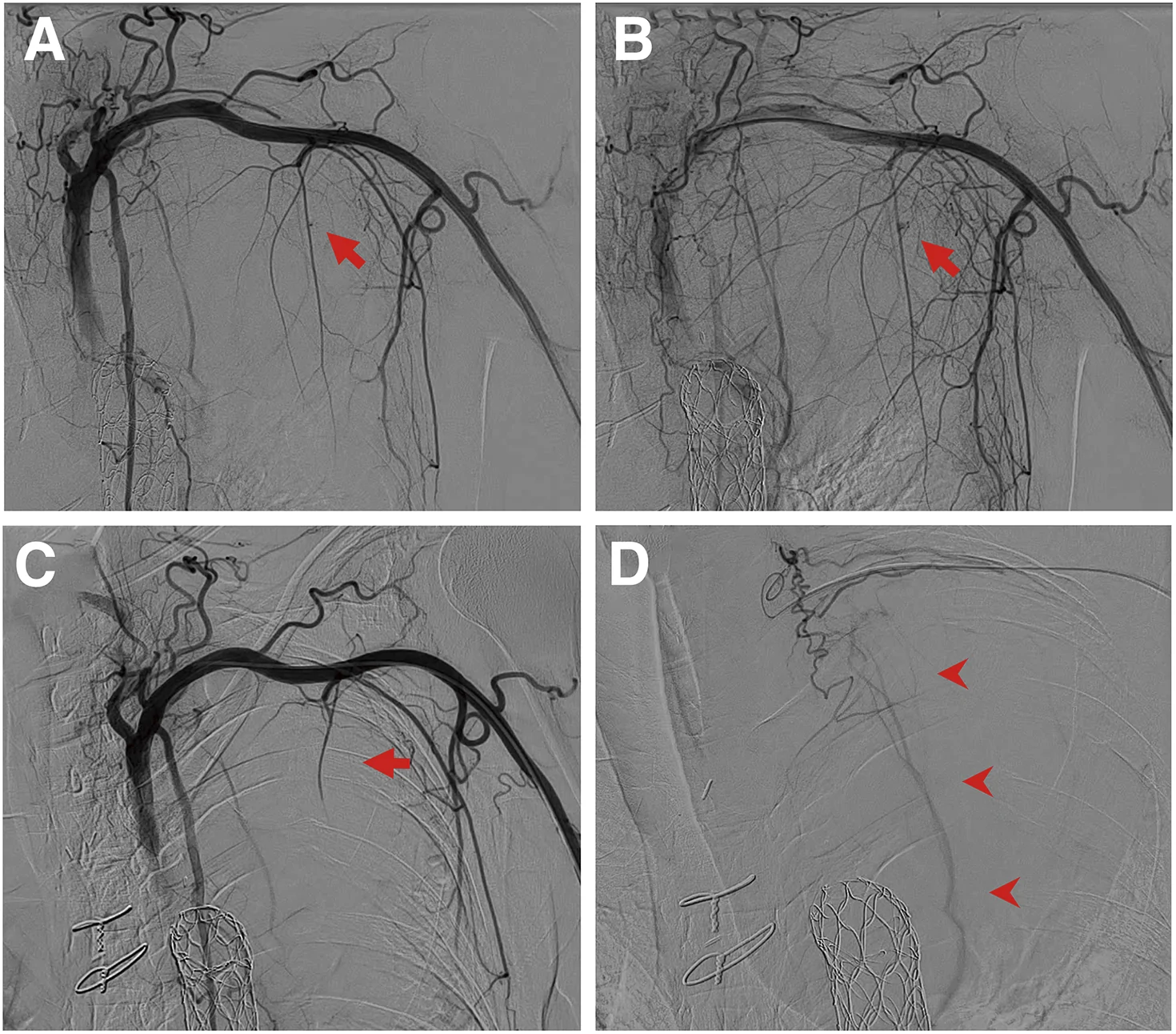
Angiographic findings. (**A**, **B**) Angiography of the left subclavian artery showing suspected bleeding from the left lateral thoracic artery (arrow). (**C**) Embolization of the left lateral thoracic artery (arrow). (**D**) Shunt flow from the chest wall arteries to the pulmonary circulation (arrowheads).

Approximately 4 months after descending aortic replacement, the patient was admitted with a massive left intrathoracic hematoma. There was no history of trauma, and the prothrombin time–international normalized ratio (PT-INR) was 2.79 while the patient was receiving warfarin anticoagulation for a mechanical valve. Chest radiography showed a large opacity in the left hemithorax (**Fig. 1A**). Contrast enhancement was observed within the hematoma on contrast-enhanced CT (**Fig. 1B**), suggesting ongoing bleeding; therefore, emergent angiography was performed. Angiography of the left subclavian artery revealed findings suggestive of hemorrhage originating from the left lateral thoracic artery (**Fig. 2A** and **2B**). However, subsequent selective angiography of the left lateral thoracic artery failed to demonstrate a pseudoaneurysm or contrast extravasation, likely because of vasospasm. As no lesion warranting embolization was identified, the procedure was terminated.

Follow-up CT performed on the following day showed enlargement of the hematoma with persistent contrast enhancement within the lesion, indicating active bleeding. Surgical intervention was considered; however, dense adhesions between the lung and chest wall were anticipated because of the patient’s previous thoracic operations. These adhesions may have helped contain the hemorrhage, whereas surgical adhesiolysis could have disrupted this containment and created additional bleeding sites. We therefore elected to repeat angiography before proceeding with surgical intervention. Although no definite bleeding source was identified on repeat angiography, given the possibility of intermittent bleeding temporarily obscured by vasospasm or minute bleeding that could not be detected angiographically, we performed empiric embolization of the suspected culprit arteries. The left lateral thoracic artery, which was suspected to be the source of bleeding on the previous angiogram, was embolized with a gelatin sponge (Serescue; Nippon Kayaku, Tokyo, Japan) via a left brachial artery approach (**Fig. 2C**). In addition, the left supreme intercostal artery, which was suspected to supply the bleeding site, was similarly embolized. Angiography via the left costocervical trunk demonstrated a shunt between the chest wall artery and the pulmonary circulation (**Fig. 2D**), but no active bleeding was identified; therefore, embolization of this shunt was not performed.

**Fig. 3:**
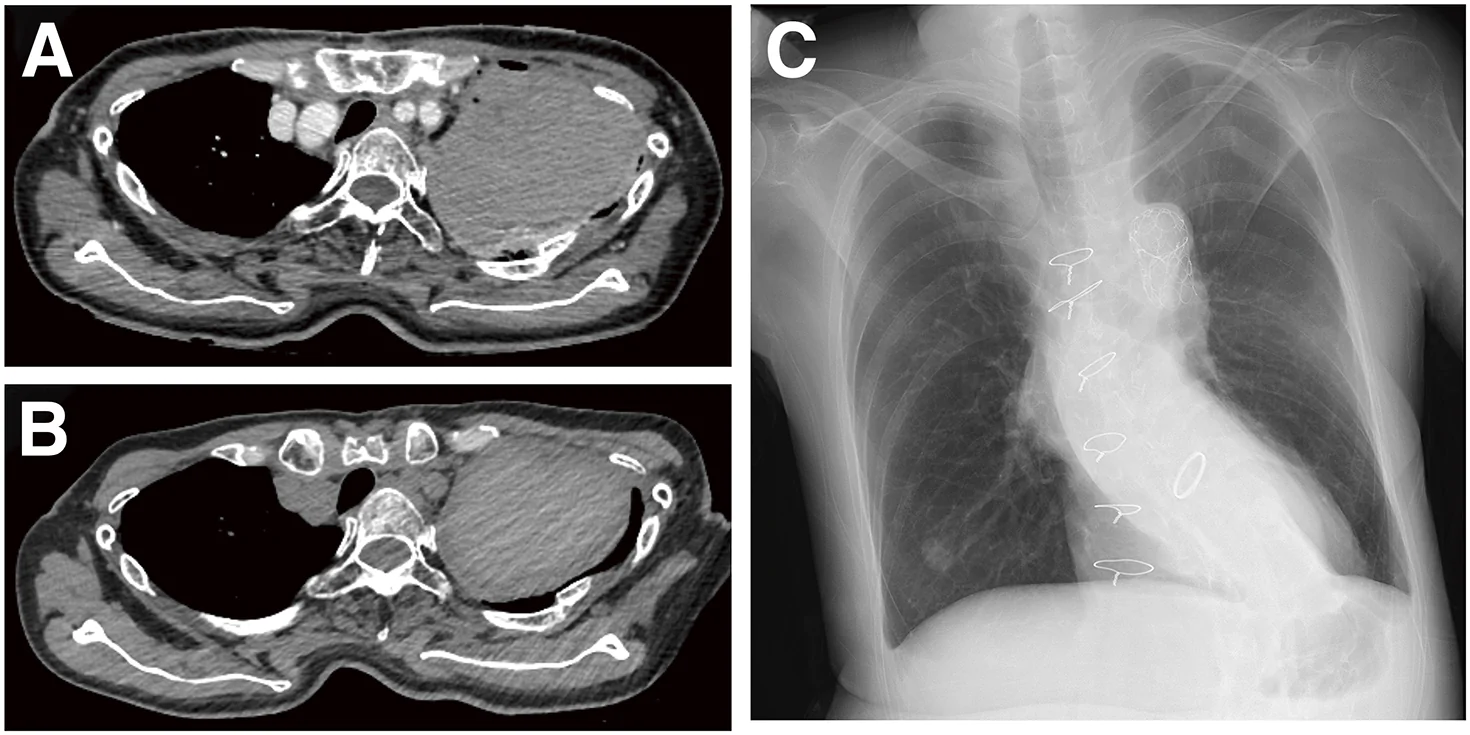
Follow-up imaging after embolization. (**A**) Contrast-enhanced CT performed 3 days after embolization showed no contrast extravasation or pseudoaneurysm. (**B**) Non-contrast CT performed 2 weeks after discharge demonstrated a reduction in the size of the hematoma. (**C**) Chest radiography at the 7-month follow-up visit demonstrated resolution of the hematoma.

Non-contrast CT performed the day after embolization showed no further enlargement of the hematoma. Furthermore, contrast-enhanced CT performed 3 days after embolization demonstrated a slight reduction in the hematoma, with no evidence of contrast extravasation or pseudoaneurysm (**Fig. 3A**). Consequently, surgical intervention was avoided. After confirming the absence of rebleeding following the resumption of anticoagulation therapy, the patient was discharged 2 weeks after embolization. Follow-up CT performed 2 weeks after discharge demonstrated a further reduction in the size of the hematoma (**Fig. 3B**). Thereafter, serial chest radiographs showed a gradual decrease in the size of the hematoma, with no recurrent bleeding or need for reintervention. At a follow-up visit approximately 7 months after discharge, chest radiography revealed resolution of the hematoma (**Fig. 3C**). Further follow-up at our institution was not possible because the patient subsequently relocated.

## Discussion

We report a patient with MFS who developed delayed hemorrhage from chest wall arteries several months after thoracic aortic surgery. To date, only a few similar cases have been described in the literature, including those reported by Yabuuchi et al. and Muraoka et al.^3,4)^ In both reports, the patients had undergone the Bentall procedure, were taking warfarin, and had excessively prolonged PT-INR values. In addition, both patients presented with hemoptysis. Our patient shared many clinical features with the previously reported cases, including MFS, prior Bentall surgery, and ongoing warfarin therapy. However, our patient did not present with hemoptysis and instead presented with left-sided chest pain and hoarseness caused by compression of the recurrent laryngeal nerve by the hematoma.

Our patient had undergone descending aortic replacement approximately 4 months earlier. However, the hematoma was located away from the surgical incision, and postoperative CT scans showed no evidence of injury to the chest wall arteries, making direct surgical injury unlikely. In a previously reported case, inflammation associated with numerous pulmonary cysts and postoperative pleural effusion was thought to have led to the release of angiogenic growth factors, resulting in abnormal anastomoses between the pulmonary and systemic circulations and subsequent bleeding from these fragile vessels.^3)^ In our patient, shunt flow between a branch of the left costocervical trunk and the pulmonary circulation was confirmed. Since there were no emphysematous changes in the lungs, we hypothesized that the inflammation associated with multiple thoracic surgeries induced angiogenesis in this case. A similar shunt involving the left lateral thoracic artery and pulmonary circulation may have formed, and the spontaneous rupture of these fragile vessels may have caused the intrathoracic hematoma. However, the actual ruptured neovessels were not directly visualized, and the precise bleeding source could not be definitively established. Therefore, bleeding from an angiographically occult small thoracic arterial branch remains an alternative explanation. Warfarin therapy may also have contributed to the severity of the hemorrhage once bleeding occurred.

Although the initial angiographic examination showed findings suspicious for extravasation, subsequent selective angiography demonstrated no evidence of bleeding, possibly because of vasospasm; therefore, embolization was considered unnecessary. However, CT performed the following day continued to demonstrate contrast material within the hematoma. Hemostasis was achieved by empiric embolization of the left lateral thoracic and left supreme intercostal arteries. In patients with bleeding in the absence of PNALs, the bleeding site may be difficult to identify, even on angiography; therefore, empiric embolization may be useful.

## Conclusions

In patients with MFS, careful CT follow-up is essential to avoid missing the optimal timing for surgical intervention. However, even in the absence of PNALs on CT, peripheral vessels may rupture spontaneously without any history of trauma. Similar cases have been reported in patients receiving warfarin after a Bentall procedure, as in our case. Hoarseness caused by recurrent laryngeal nerve compression may be an important clinical indicator of intrathoracic hematoma formation. Therefore, even in the absence of hemoptysis, intrathoracic bleeding should be considered in patients with lateral chest pain accompanied by hoarseness. When the bleeding site is difficult to identify in such cases, empiric embolization may be a useful therapeutic option.

## References

[1] Judge DP, Dietz HC. Marfan’s syndrome. Lancet 2005; 366: 1965–76. 16325700 10.1016/S0140-6736(05)67789-6 PMC1513064

[2] Sénémaud J, Gaudry M, Jouve E, et al. Primary non-aortic lesions are not rare in Marfan syndrome and are associated with aortic dissection independently of age. J Clin Med 2023; 12: 2902. 37109238 10.3390/jcm12082902 PMC10141376

[3] Yabuuchi Y, Goto H, Nonaka M, et al. A case of Marfan syndrome with massive haemoptysis from collaterals of the lateral thoracic artery. BMC Pulm Med 2020; 20: 4. 31914988 10.1186/s12890-019-1033-1 PMC6951026

[4] Muraoka T, Suzuki K, Kuramochi M, et al. 3 cases of lateral thoracic artery lesions that required endovascular or surgical treatment. Jpn J Vasc Surg 2023; 32: 405–9. (in Japanese)

